# ES-mediated chimera analysis revealed requirement of DDX6 for NANOS2 localization and function in mouse germ cells

**DOI:** 10.1038/s41598-018-36502-0

**Published:** 2019-01-24

**Authors:** Ryuki Shimada, Makoto Kiso, Yumiko Saga

**Affiliations:** 1 Department of Genetics, SOKENDAI, Yata 1111, Mishima, Shizuoka, 411-8540 Japan; 2 Research Fellow of Japan Society for the Promotion of Science, Tokyo, Japan; 3 Division of Mammalian Development, Genetic Strains Research Center, Yata 1111, Mishima, Shizuoka, 411-8540 Japan; 4 Mouse Research Supporting Unit, National Institute of Genetics, Yata 1111, Mishima, Shizuoka, 411-8540 Japan; 5 Department of Biological Sciences, Graduate School of Science, The University of Tokyo, Tokyo, 113-0033 Japan

## Abstract

In embryonic male germ cells, the RNA-binding protein NANOS2 recruits its target RNAs to processing bodies (P-bodies), where they are repressed. This process is necessary to promote male-type germ cell differentiation. However, it remains unclear whether all NANOS2 functions depend on P-bodies. To address this question, we established ES cell lines containing a germ cell-specific inducible Cre and reporter together with the floxed *Ddx6* allele. We deleted the *Ddx6* gene by administering tamoxifen to chimeric embryos containing germ cells derived from recombinant ES cells. DDX6-null germ cells exhibited both similar and distinct defects from those observed in NANOS2-null germ cells. These results demonstrate that NANOS2 function is carried out via both P-body-dependent and -independent mechanisms. RNA-seq analyses further supported the phenotypic differences between DDX6-null and NANOS2-null germ cells, and indicated distinct molecular cascades involved in NANOS2-mediated gene regulation.

## Introduction

Germ cells are specialized cells required for transmitting genetic information to the next generation. In mice, primordial germ cells (PGCs) are segregated from the somatic cell lineage at E7.25 and proceed to migrate to the future gonads^1^. After colonizing the embryonic gonads with somatic cells, PGCs start sexual differentiation depending on the environment. In the ovary, retinoic acid (RA) derived from the mesonephros triggers the expression of the meiosis initiator gene *Stimulated by RA gene 8* (*Stra8*), which causes the germ cells to enter meiotic prophase. On the other hand, in male gonads, RA signaling is inhibited by the RA degrading enzyme CYP26B1 produced by somatic cells^2,3^, and germ cells enter mitotic arrest. In addition, NANOS2 expression starts at around E13.5 in male germ cells, which represses meiosis and promotes male-type differentiation^4^.

Nanos is an evolutionarily conserved RNA binding protein that exists in both invertebrates and vertebrates^5–7^, and was demonstrated to function as a posttranscriptional gene repressor. Among the three *Nanos* genes in mice, NANOS2 plays a key role in male germ cell development^4–8^. Male germ cells enter G1-G0 arrest before NANOS2 expression starts, but NANOS2-null germ cells fail to maintain this G0 state and resume mitotic activity. Furthermore, many NANOS2-null germ cells ectopically express STRA8 and initiate meiosis even in the male gonad. The effects of NANOS2 are not limited to the suppression of meiosis, as it also promotes male-type gene expression. NANOS2-null germ cells fail to express DNMT3L, one of the epigenetic regulators important for DNA methylation, including genomic imprinting^9–11^. Thus, these NANOS2-null phenotypes may be due to the upregulation of NANOS2 target genes. NANOS2-null germ cells exhibit several other phenotypes. For example, the expression of another Nanos protein, NANOS3, is upregulated^12^ even though *Nanos3* is not a direct target of NANOS2. Moreover, some germ cells are abnormally located in the interstitial space of seminiferous tubules in the absence of NANOS2^13^. However, the molecular mechanisms underlying these abnormal phenotypes are unknown.

Previous studies have reported that NANOS2 protein interacts with the CCR4-NOT deadenylation complex^12,14,15^ and localizes to P-bodies. P-bodies are messenger ribonucleoprotein (mRNP) granules, which contain components of mRNA decay machinery, such as DCP1/DCP2 decapping enzyme and the 5′ to 3′ exonuclease XRN1^16–18^, implying that P-bodies are the centers of mRNA decay. We therefore expect that decapping and 5′-3′ exonucleolytic decay of NANOS2-target mRNAs occurs following deadenylation by CCR4-NOT in P-bodies^19–22^. However, it remains unclear whether P-bodies are required for NANOS2 function, and if so, whether all NANOS2 functions are P-body-dependent.

To clarify this issue, we aimed to disrupt P-body formation and analyze the resulting phenotypes. Some previous reports demonstrated that P-body loss can be caused by the depletion of some P-body components^16,17,23,24^. Among these components, we focused on DDX6 (Rck/p54), which is a core component of P-body assembly. DDX6 (also known as Me31b in flies and Dhh1 in yeast) is a DEAD-box protein with ATPase/helicase activity. Although no *in vivo* knockout study has been reported, *Ddx6*-KO is expected to be embryonic lethal because of the importance of DDX6-mediated RNA regulation in development. Therefore, we planned to generate conditional KO (cKO) mice. In general, ES-derived chimeric mice are used to generate cKO mouse lines. This line has to be crossed with a germ cell-specific Cre line to establish a male mouse with Cre. Then, the male floxed-*Ddx6*/Cre mouse can be crossed with a homozygous floxed female mouse. Therefore, it takes more than 6 months to obtain cKO embryos even using the quickest protocol. Furthermore, in the case of germ cell studies, we only focus on males or females depending on the purpose (male in our case). As such, only 1/8 can be expected to be male cKO embryos. Thus, we need to cross many mice to increase the efficiency. However, we can solve many problems by establishing a good ES cell line for chimera production, and ideally obtain cKO results within 2 weeks.

We demonstrate here that the chimera method is highly effective. Using this method, we generated chimeric embryos that contain DDX6-null germ cells. The results strongly support the recruitment of NANOS2 in P-bodies being a critical step to repress target RNA. On the other hand, NANOS2 may also work in a DDX6- and P-body- independent manner.

## Result

### Establishment of useful *mT/mG*;*Oct4dPE-creER*^*T2*^ ES line for chimeric analysis of germ cell development

**Figure 1:**
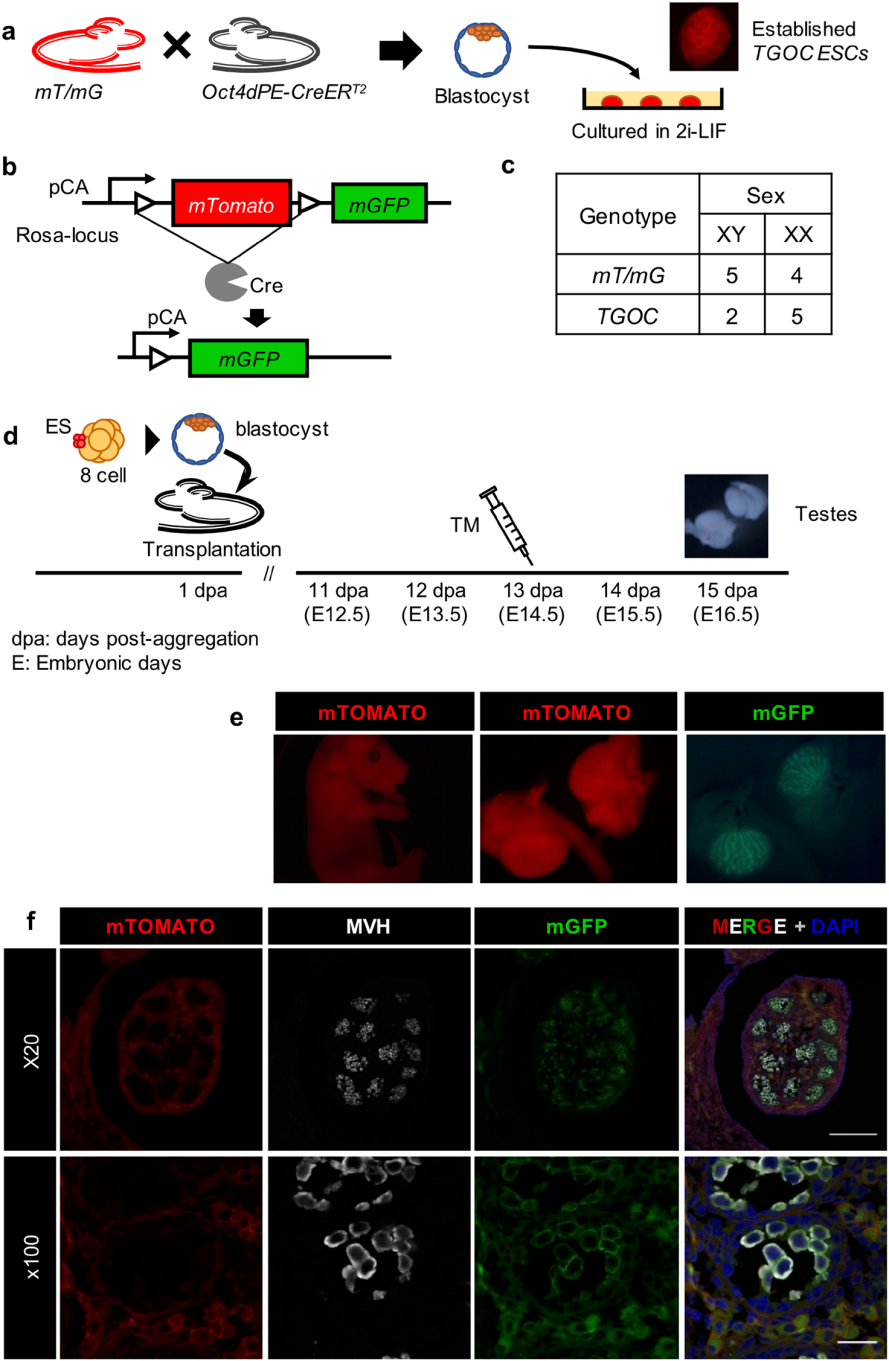
Establishment of ES lines and chimeric analyses. (**a**–**c**) ES cell lines were established by cultivating blastocysts prepared from intercrossed mothers of *Oct4dPE*-*CreER*^T2^ and Rosa-*mT*/*mG*. Established TGOC ESCs expressed mTOMATO (**a**). Before Cre mediated recombination, the chicken β-actin core promoter with the *CMV* enhancer (*pCA*) drives mTOMATO expression. After Cre recombination, the *mTomato* sequence is excised, and *pCA* drives mGFP expression (**b**). See Fig. S2. (**c**) List of established ESC-lines. We obtained 16 lines: 5 male and 4 female *mT/mG* ES lines, and 2 male and 5 female TGOC ES lines. (**d**) Scheme of the experimental procedure for chimera analyses. ESCs were aggregated with 8-cell embryos and the formed blastocysts were transferred to a foster mother (1 dpa). To induce Cre activity, tamoxifen (TM) was administered at an appropriate stage and testes were collected from 15-dpa (E16.5) embryos. See Fig. S1. (**e**) Fluorescence images of a representative chimera and testes produced by TGOC ES cells with mTOMATO (red) and mGFP (green). TM was administered at 13 dpa and the chimeric embryo was recovered at 15 dpa. (**f**) Fluorescence images for mTOMATO, and immunohistochemistry for the germ cell marker MVH (magenta) and mGFP of testis sections are shown in (**e**). Scale bar in x20 image, 150 µm; x100 image, 25 µm.

To establish ES lines suitable for chimera analyses in a germ cell-specific manner, we crossed *Oct4dPE-CreER*^*T2*^ and Rosa-*mT/mG* mice (Fig. 1a). As *Oct4dPE-CreER*^T2^ mice express the tamoxifen-inducible Cre recombinase under the control of the *Oct-dPE* promoter-enhancer, its expression is restricted to germ cells after E9.5^25^. The *mT/mG* mice globally express a membrane-targeted version of tdTomato (*mT*/mTOMATO) and the expression changes to the membrane-targeted version of EGFP (*mG*/mGFP) after recombination via Cre activity^26^ (Fig. 1b). Reporter incorporation is important for chimera analyses because we have to distinguish ES-derived cells (mTOMATO or mGFP) from wild-type host cells. In addition, we can mark Cre-active cells by mGFP expression. Blastocysts obtained from two female mice were separately cultured in 2i-LIF medium and we established 16 ES cell lines; 5 XY and 4 XX *mT/mG*, and 2 XY and 5 XX *m****T****/m****G***;***O****ct4dPE-****C****reER*^*T2*^ (we referred to this genotype as TGOC) (Fig. 1c).

Using one of the XY TGOC ES lines, we produced chimera to check the ability of the ES line to contribute to germ cells in chimera and whether we can induce germ cell-specific Cre activation via tamoxifen injection. We employed the ES aggregation method using 8-cell embryos to produce the chimeras (see the methods section), and orally administered tamoxifen (TM) to the foster mother at 13 days post-aggregation (dpa, which stage corresponds to E14.5) to induce the CreER^T2^ activity. We dissected the foster mother to recover chimeric embryos at 15 dpa (corresponding to E16.5) (Fig. 1d). We first selected chimeras by black eye color because ES cells originated from colored mice were aggregated with 8 cell embryos from white mice (MCH). We confirmed that most of the embryos with black eyes were males due to the higher contribution of XY-ES cells, as expected (Fig. S1). Most chimeric embryos had strong mTOMATO signal throughout the body, but mGFP signal was observed only in the gonads (Figs S1, 1e). The gonad sections were prepared and immunostained for GFP and the germ cell marker MVH. As a result, only germ cells detected by MVH were positive for mGFP, indicating that Cre recombination was correctly induced only in germ cells (Fig. 1f). Therefore, we decided to use this TGOC ES line for further experiments.

Maintaining ESCs in a good condition is critical for chimera analyses. Once Cre recombinase is activated, it is impossible to perform conditional KO. We found that some ESCs started to express GFP during ES culture (Fig. S2A). To find the appropriate conditions for ESC maintenance, we compared serum-free medium (2i-LIF) with serum-containing medium (serum+). Even when ESCs were cultured in 2i-LIF, approximately 5% of the ESCs exhibited GFP expression, but this population increased to 17% when they were cultured in serum + medium (Fig. S2B). Thus, we maintained the ESCs in serum-free 2i-LIF medium.

### Establishment of the floxed-*Ddx6* TGOC ES line using CRISPR/Cas9

**Figure 2:**
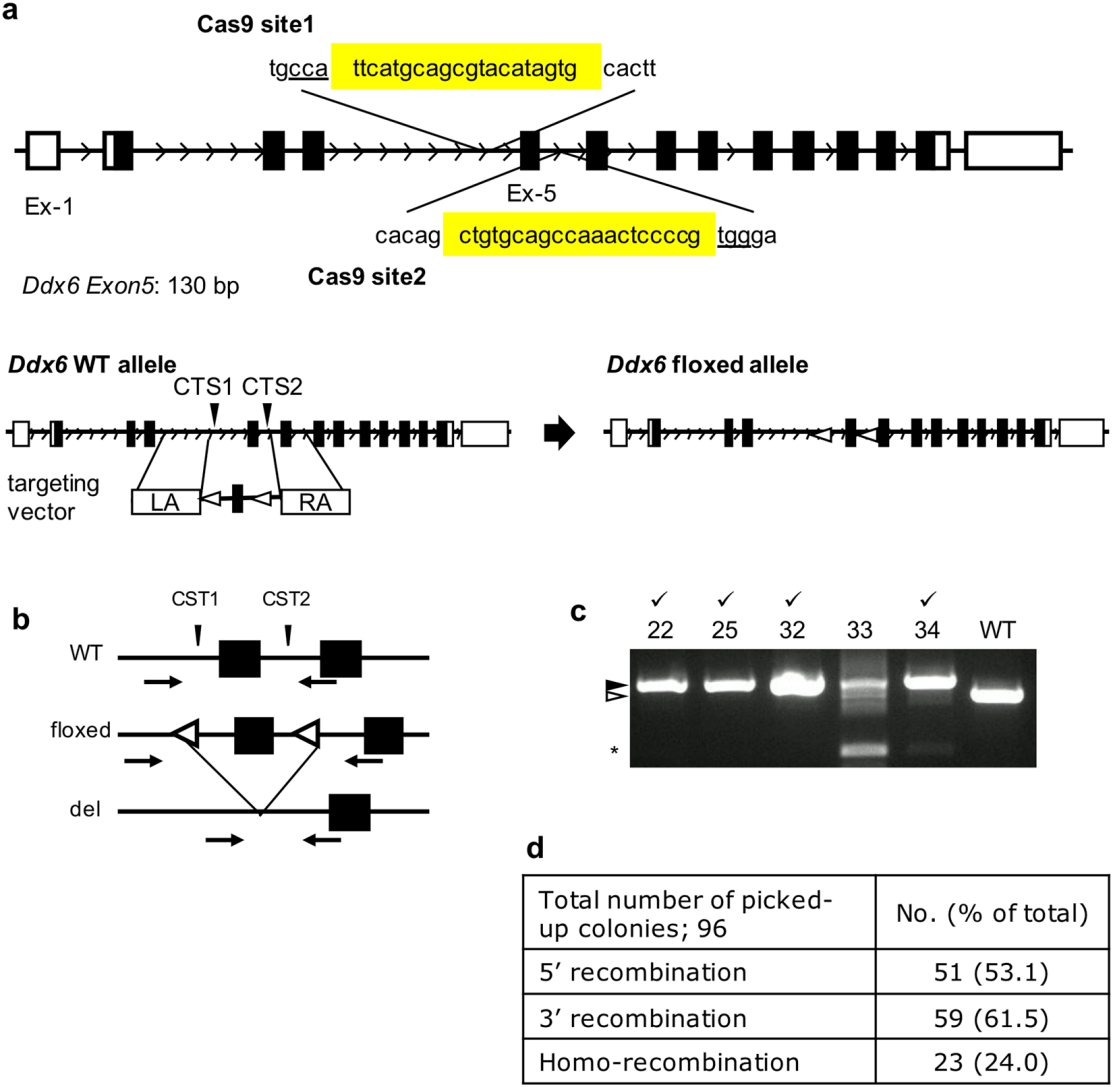
Strategy to generate the *Ddx6*-cKO allele using CRISPR/Cas9. (**a**) Gene structure of the WT *Ddx6* allele and *floxed-Ddx6* allele induced by homologous recombination with a targeting vector. Two Cas9 sites were designed to flank exon 5 (130 bp). White boxes indicate the UTR region, black boxes indicate the CDS-encoding exons and white triangles indicate the lox-p sequences. (**b**) ESC genotyping strategy. Arrows indicate the primers that can distinguish the WT, floxed and deleted *Ddx6* alleles. (**c**) Genotyping results of several ES clones. ES clones in which *floxed* alleles were introduced in both chromosomes are indicated. The black arrowhead indicates the *floxed*-allele, white arrowhead indicates WT and the asterisk indicates the deleted allele. (**d**) Efficiency of homologous recombination by 5′- and 3′-specific PCR primers.

To conditionally KO the *Ddx6* gene, we introduced the floxed-*Ddx6* allele to the TGOC line. For this purpose, we took advantage of CRISPR/Cas9 mediated homologous recombination. We designed two Cas9 vectors for two target sites to remove *Ddx6*-exon5, and a targeting vector containing floxed exon-5 (Fig. 2a). Removal of exon5 leads to a frame-shift and produces a nonfunctional protein. We transfected these three constructs into TGOC ES cells and obtained ES cell clones after puromycin selection. The genotypes of ES clones were determined by primer pairs that can distinguish WT, ex-5 floxed, and ex-5 deleted alleles (Fig. 2b). Several ES lines had *floxed-Ddx6* alleles introduced in both chromosomes (Fig. 2c,d).

**Figure 3:**
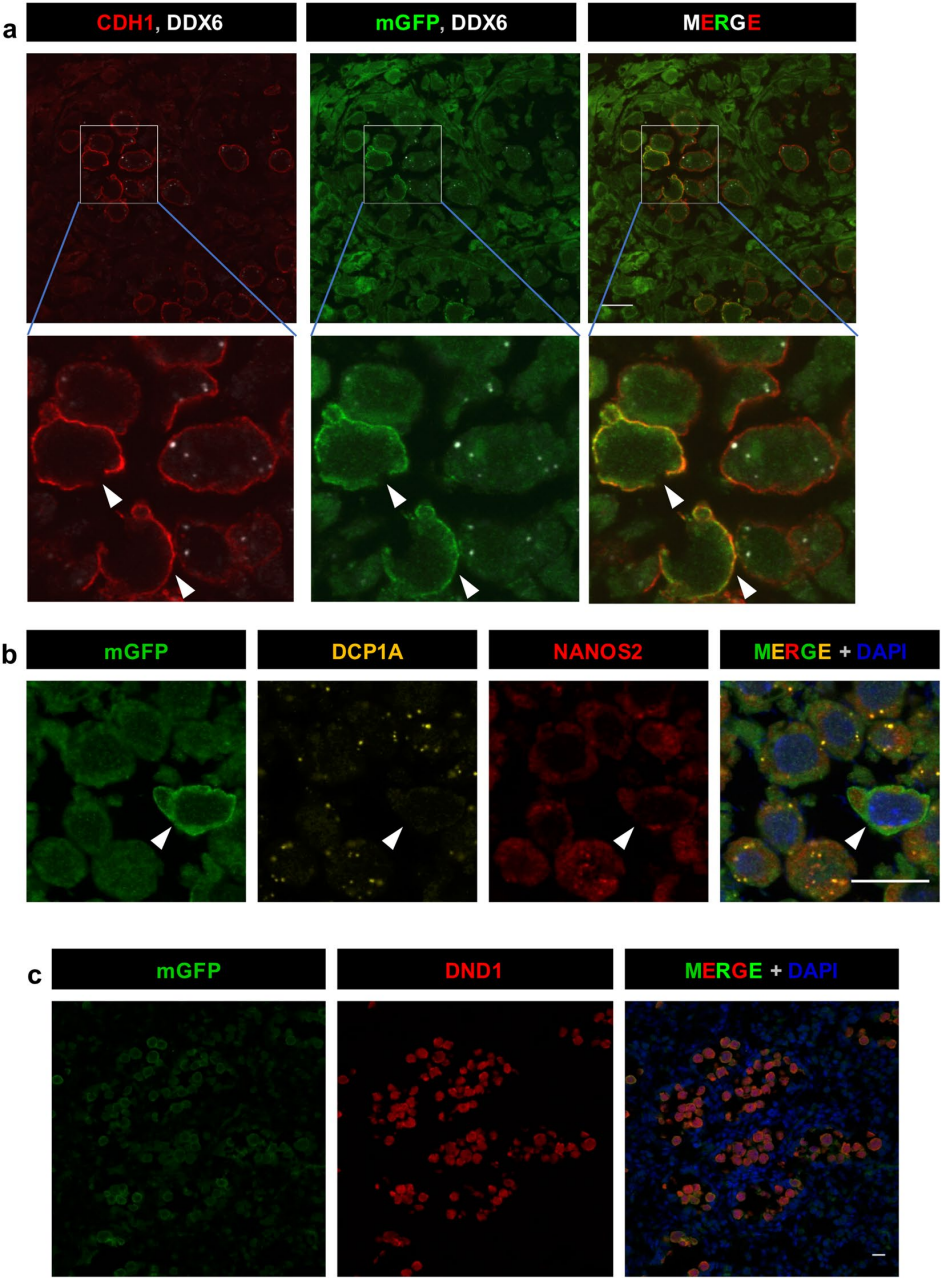
*Ddx6*-KO leads to P-body loss in germ cells. (**a**) Immunofluorescence images of chimeric testes. mGFP-positive cells lost DDX6 foci. CDH1 (red) was used as a germ cell marker; DDX6 is shown in white and GFP is shown in green. Scale bar indicates 20 µm. (**b**) Immunohistochemistry for DCP1A (yellow) and NANOS2 (red). (**c**) Immunohistochemistry for DND1 (red). Arrows indicate the GFP-positive DDX6-null germ cells. Scale bars indicate 15 µm.

Using the established floxed-*Ddx6* TGOC ES cell lines, we produced chimeras and evaluated whether *Ddx6* was correctly knocked-out in the germ cells. In this experiment, we injected tamoxifen at 11 dpa (corresponding to E12.5) before formation of NANOS2-P-body foci. In chimeric gonads, both mGFP-positive and mGFP-negative germ cells were observed, and mGFP-positive germ cells had lost DDX6 foci on immunostaining with the anti-DDX6 antibody, whereas mGFP-negative germ cells contained clear DDX6 foci (Fig. 3a). In addition, we found that DDX6-null germ cells lost DCP1A foci, which is a marker of P-bodies (Fig. 3b). Thus, we successfully depleted P-bodies in a germ-cell-specific manner by *Ddx6*-cKO. Of note, NANOS2 was still expressed in the cytoplasm of DDX6-null cells but was not merged with DCP1A in discrete foci, indicating that NANOS2 foci are not generated without DDX6 (Fig. 3b). In addition to NANOS2 expression, an essential partner of NANOS2^13^, DND1, was also detected in DDX6-null germ cells (Fig. 3c). This suggests that NANOS2 can make a functional complex with DND1 even in the absence of DDX6. An important question is whether NANOS2 can function normally without forming P-bodies. We previously produced *Nanos2*-KO mice^27^. However, to rule out effects from the chimera analysis and tamoxifen injection, we also established *Nanos2*-KO TGOC ES lines using CRISPR/Cas9 (Fig. S3A). Similar to the *Ddx6-*cKO, *Nanos2*-KO germ cells were also correctly marked by mGFP after TM administration in chimeric embryos (Fig. S3B).

### DDX6-dependent P-body formation is required for NANOS2 to repress target gene expression

DDX6 is implicated in RNA regulation by functioning as a scaffold protein to assemble P-bodies^24^. Therefore, DDX6-null germ cells may have several defects. However, if all functions of NANOS2 depend on P-bodies, DDX6-null germ cells should exhibit all the phenotypes of *NANOS2*-null germ cells. To clarify this hypothesis, we compared DDX6-null germ cells with NANOS2-null germ cells for the following NANOS2-downstream phenotypes; (1) up-regulation of the NANOS2 target *Dazl*, (2) loss of DNMT3L expression, (3) resumption of mitosis, (4) up-regulation of STRA8, indicating meiosis initiation, and (5) germ cells escaping from the seminiferous tubules.

**Figure 4:**
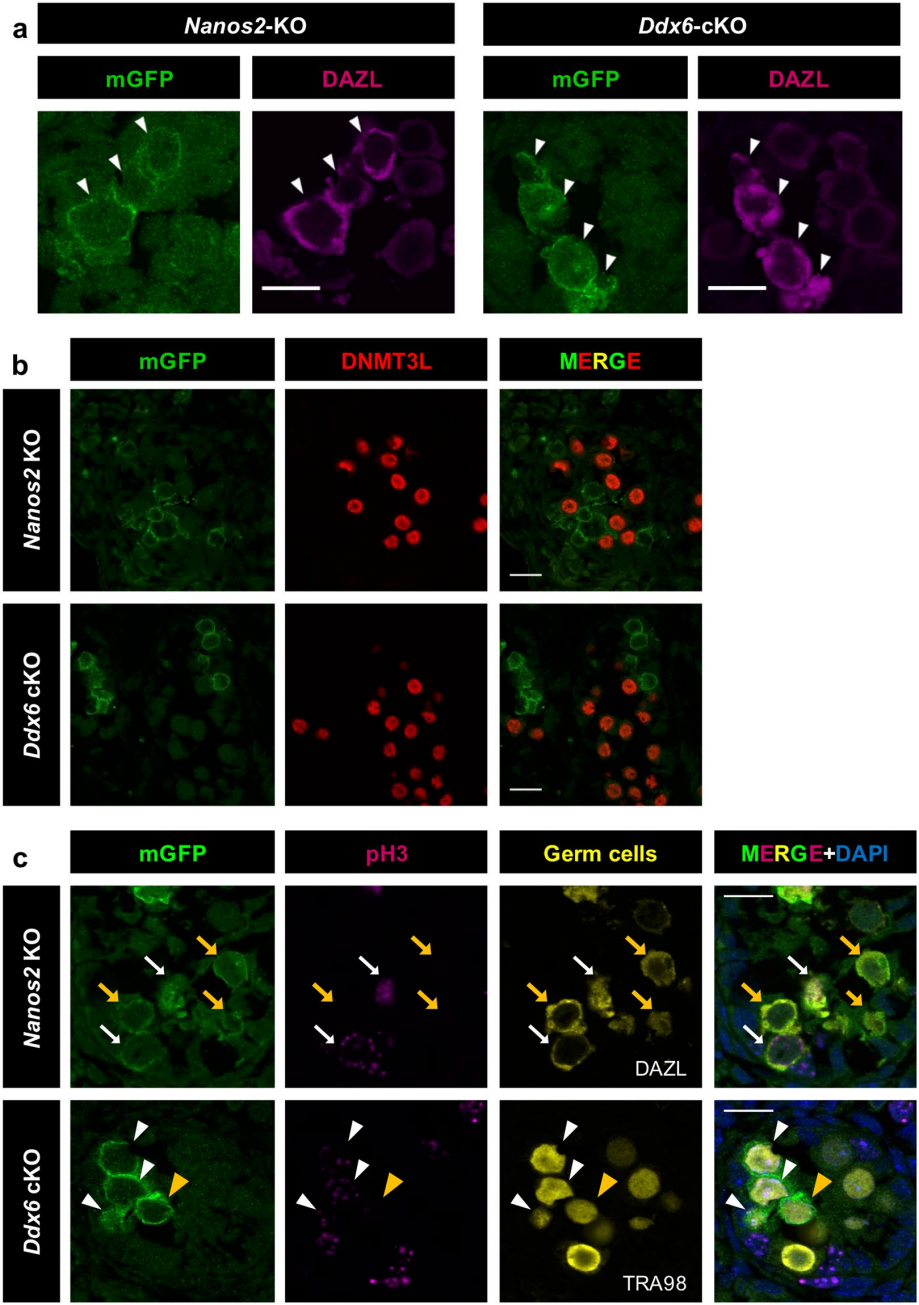
DDX6-null germ cells and NANOS2-null germ cells exhibited similar phenotypes. Immunofluorescence images of testis sections prepared from chimeric embryos with either *Nanos2*-KO or *Ddx6*-cKO ES cells. See Fig. S3 for the *Nanos2*-KO strategy. mGFP-positive cells represent ES-derived gene-KO cells. Sections were stained with anti-DAZL (magenta) (**a**), DNMT3L (red) (**b**) and anti-pH3 (magenta) (**c**) antibodies. To identify germ cells, anti-DAZL(a) or anti-TRA98 (yellow in **c**) antibodies were used; *Nanos2*-KO (n = 3), *Ddx6*-cKO (n = 4). White arrows indicate the GFP-positive, NANOS2-null, pH3-positive germ cells. Orange arrows indicate the GFP-positive, NANOS2-null, pH3-negative germ cells. White arrowheads indicate the GFP-positive, DDX6-null, pH3-positive germ cells. Orange arrowheads indicate the GFP-positive, pH3-negative germ cells. Scale bars are 15 µm.

The major function of NANOS2 is proposed to be the degradation of target RNA in P-bodies, and one of the identified target genes is *Dazl*^28^. Therefore, in the absence of NANOS2, DAZL accumulates due to mRNA stabilization. To evaluate whether NANOS2 functions normally in the absence of P-bodies, we performed immunofluorescence staining using anti-DAZL antibody. Consistent with the previous study, DAZL expression was significantly increased in NANOS2-null germ cells even in chimeras (Fig. 4a). Similarly, DDX6-null germ cells also had up-regulation of DAZL expression (Fig. 4a). This result strongly suggests that NANOS2 cannot suppress DAZL expression in the absence of P-bodies.

Next, we investigated the promotion of male germ cell development by NANOS2 by examining DNMT3L. DNMT3L induces the male-type epigenetic state in cooperation with the DNA-methyltransferases DNMT3A and 3B. Thus, lack of DNMT3L leads to defective spermatogenesis^10,11^. In the chimeras, the majority of WT germ cells highly expressed DNMT3L (Fig. 4b), whereas both NANOS2-null and DDX6-null germ cells lacked DNMT3L expression (Fig. 4b). This indicates that NANOS2 cannot promote proper male germ cell development without P-bodies. To further examine male germ cell development, we confirmed the cell cycle state using pH3 staining. It is known that male germ cells enter cell cycle arrest after E14.5 and the arrested state is maintained until cell proliferation resumes just after birth. In mGFP-positive germ cells in both *Nanos2*-KO and *Ddx6*-cKO chimeric embryos, pH3 signals were clearly detected, demonstrating that cell cycle arrest cannot be maintained in DDX6-null or NANOS2-null germ cells^4^ (Fig. 4c). Thus, P-bodies may be required for NANOS2 to promote male germ cell development. We concluded that NANOS2 is unable to function efficiently in the absence of P-bodies because germ cells lacking DDX6 and NANOS2-null germ cells had similar phenotypes.

### NANOS2 may also function in a P-body-independent manner

**Figure 5:**
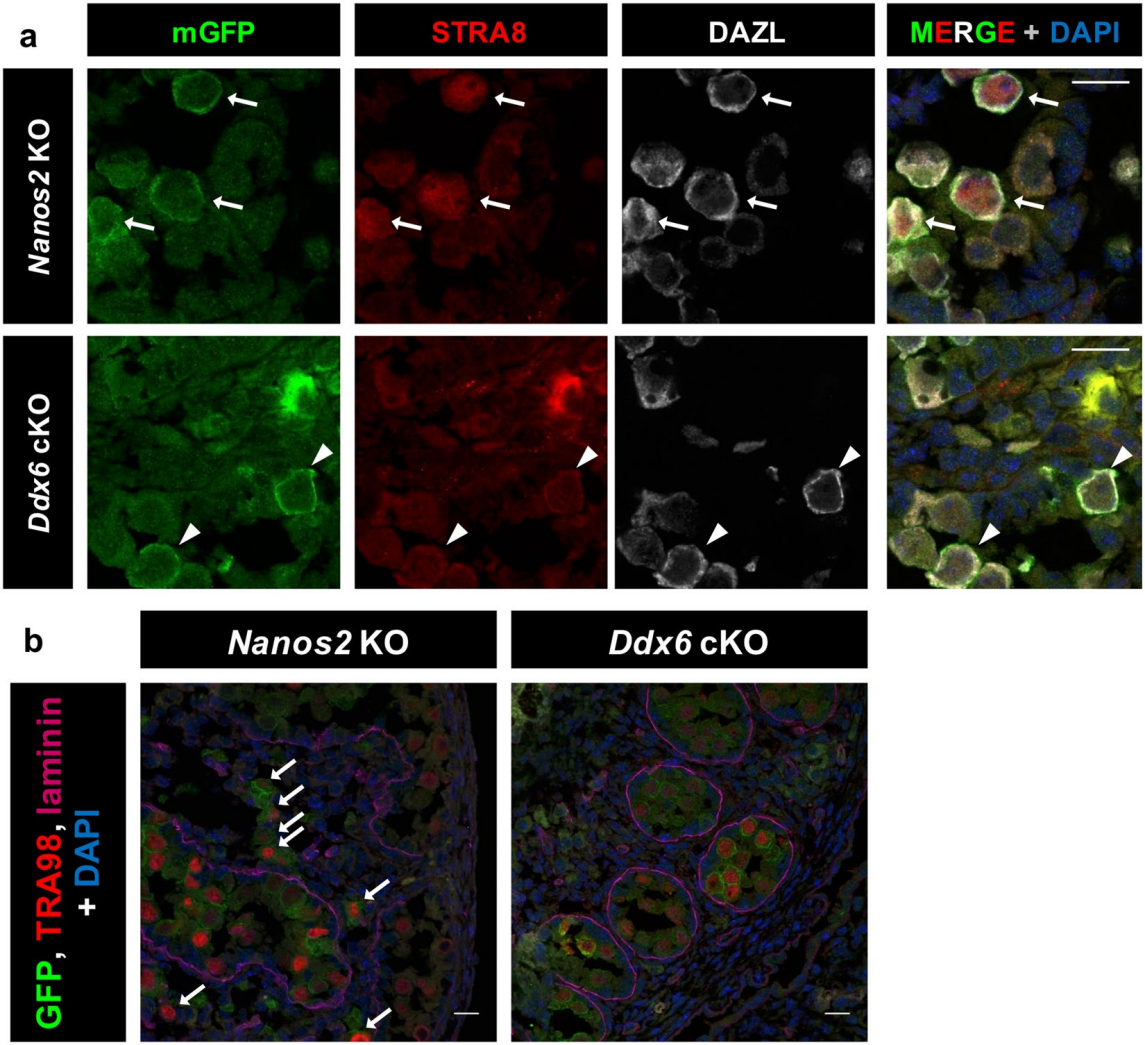
DDX6*-*null germ cells also had different phenotypes from NANOS2-null germ cells. (**a**,**b**) Immunofluorescence images of testis sections prepared from chimeric embryos with either *Nanos2*-KO or *Ddx6*-cKO ES cells. mGFP-positive cells indicate ES-derived gene-KO cells. Sections were stained with anti-STRA8 (red) (**a**) and anti-laminin (magenta) antibodies (**b**). To identify germ cells, anti-DAZL (**a**) or anti-TRA98 (red) (**b**) antibodies were used. DAPI was used to stain DNA. *Nanos2*-KO (n = 3), *Ddx6*-cKO (n = 4). Scale bar indicates 15 µm. Arrows indicate germ cells that escaped from the seminiferous tubules.

In addition to resumption of the cell cycle, some NANOS2-null germ cells entered meiosis^8^. In mice, meiosis is induced by RA, and expression one of the RA responsive genes, STRA8, is used as a marker of meiosis initiation in embryonic ovaries^29^. In male gonads, STRA8 expression is repressed because of low RA levels and the repressive function of NANOS2^4^. STRA8 is expected to work as a transcription factor and localizes to the nucleus to trigger meiosis^4,30,31^. Consistent with the previous study, NANOS2-null germ cells had nuclear STRA8 signals (Fig. 5a). However, such STRA8 expression was not detected in DDX6-null germ cells (Fig. 5a), suggesting that meiosis was not initiated in the DDX6-null germ cells.

We also focused on another abnormal phenotype of NANOS2-null germ cells. In male gonads, all germ cells are enclosed within an organized structure, the seminiferous tubules. However, NANOS2-null germ cells are often observed outside of these tubules, suggesting a strange escaping behavior. We performed laminin staining to mark the outer surface of the seminiferous tubules and examined whether DDX6-null germ cells also escaped. Consistent with previous observations, NANOS2-null germ cells escaped from tubules even in the chimeras (Fig. 5b), indicating that this escaping behavior is a cell autonomous defect induced by the lack of NANOS2. On the contrary, we did not observe any escaping DDX6-null germ cells (Fig. 5b). Therefore, some functions of NANOS2 are carried out independent of DDX6-mediated P-body formation.

### Global transcriptome changes between NANOS2*-*null and DDX6-null germ cells

**Figure 6:**
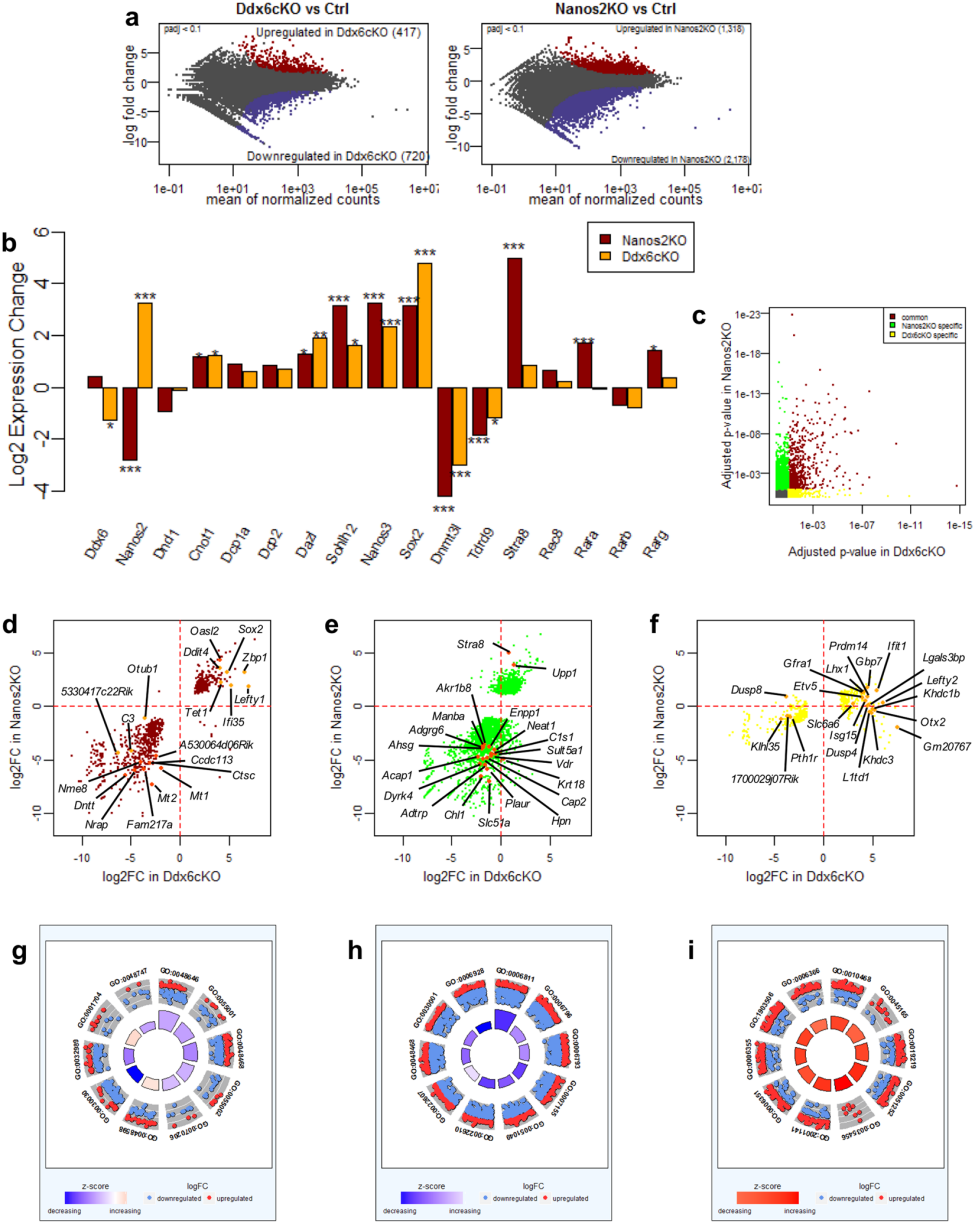
Transcriptome analysis for *Nanos2*-KO and *Ddx6-*cKO germ cells. (**a**) MA plot for *Ddx*6-cKO vs Ctrl and *Nanos2*-KO vs Ctrl. In each plot, red dots indicate the upregulated genes and blue dots indicate downregulated genes in each mutant. See Fig. S4 for FACS sorting of each germ cell genotype. (**b**) Log2 fold changes (log2FCs) of selected genes are presented. Red and yellow bars indicate log2FCs of each gene in *Nanos2*-KO and *Ddx6*-cKO, respectively. *P < 0.05, **P < 0.01 and ***P < 0.001. (**c**) Adjusted P-value comparison between *Nanos2*-KO and *Ddx6*-cKO. Red dots indicate genes whose adjusted P-value (padj) was below 0.1 in both mutants (common DEGs), green dots indicate genes whose padj was below 0.1 only in *Nanos2*-KO (*Nanos2*-KO specific DEGs) and yellow dots indicate genes whose padj was below 0.1 only in *Ddx6*-cKO (*Ddx6*-cKO specific DEGs). (**d**–**f**) Log2FCs of common (**d**), *Nanos2*-KO-specific (**e**) and *Ddx6*-cKO-specific (**f**) DEGs are shown. The top 20 affected genes in each group are indicated in orange or dark orange, respectively, with gene names. (**g**–**i**) The top 10 GO IDs (biological processes) for common (**g**), *Nanos2*-KO-specific (**h**) and *Ddx6*-cKO-specific (**i**) DEGs are presented. The outer circle is the scatter plot for each term assigned for the log2FC genes. Red and blue dots indicate up- and down-regulated genes, respectively. The inner circle represents the adjusted P-value and z-score. See Fig. S5.

To more comprehensively examine the difference between DDX6-null and NANOS2-null germ cells, we compared their transcriptomes. As KO germ cells are labeled by EGFP, we used fluorescence-activated cell sorting (FACS) to collect them from chimeric embryos (Fig. S4). We also collected EGFP-positive germ cells from chimeras generated by parental ES cells as a control. We first compared *Nanos2*-KO and *Ddx6*-KO data with control data, and found that the expression of many genes changed in both genotypes (Fig. 6a). Consistent with our immunostaining data, the expression of *Nanos2*, *Dnd1* and *Cnot1* was not downregulated in *Ddx6*-cKO germ cells. This confirmed that in DDX6-null germ cells, NANOS2 can make a complex with its known interactors. Moreover, in accordance with previous microarray and RNA-seq data using whole gonads^28,32^, NANOS2 target genes, *Dazl* and *Sohlh2*, and other genes, *Stra8*, *Nanos3* and *Sox2*, were upregulated, and male marker genes, *Dnmt3l* and *Tdrd9*, were down-regulated in *Nanos2*-KO germ cells (Fig. 6b). The expression of *Ddx6* and known P-body components, *Dcp1a* and *2*, was not significantly different in *Nanos2*-KO germ cells, but *Cnot1* was significantly upregulated in germ cells from both mutants (Fig. 6b). Although most gene expression changes were also observed in *Ddx6*-cKO germ cells, *Stra8* was not significantly upregulated (Fig. 6b), consistent with our IF data, as shown in Fig. 5a. In addition to *Stra8*, the RA receptor genes *Rarα* and *Rarγ* were highly up-regulated only in *Nanos2*-KO germ cells (Fig. 6b), suggesting that a DDX6-independent mechanism is involved in the RA-related gene regulation by NANOS2.

To search for possible genetic cascades responsible for the DDX6- or P-body- dependent or independent NANOS2 functions, we classified gene sets common to *Nanos2*-KO and *Ddx6*-cKO (common DEGs), and those only in *Nanos2*-KO (*Nanos2*-KO specific) or *Ddx6*-cKO (*Ddx6*-cKO specific), and genes that were up or down-regulated in each case were plotted (Fig. 6c–f). To further characterize these gene sets, we performed Gene Ontology (GO) analyses. The results were visualized using R package GOplot^33^, and the top 10 GO terms with representative genes are described (Figs 6g–i and S5A–C). Common GO terms comprised genes involved in muscle development and many biological processes involved in embryogenesis, many of which were downregulated (Fig. S5A). We also noted distinct GO terms for *Nanos2*-KO and *Ddx6*-cKO germ cells, respectively. Specific terms observed for *Ddx6*-cKO germ cells were gene expression related, such as transcription and RNA metabolism, and most included genes were up-regulated, suggesting that DDX6 suppresses global transcription and translation in germ cells (Fig. S5C). This is reasonable because P-body loss results in the de-repression of global gene expression. In addition, genes enriched in PGCs or early male germ cells, such as *Prdm14* and *Otx2*, were listed. This suggests that DDX6-null germ cells fail to initiate the male pathway and remain in a pluripotent state compared with normal and NANOS2-null germ cells. On the other hand, in the case of *Nanos2*-KO, many of the categories were related to ion transport and signaling, and the included genes were often downregulated, indicating structural and functional changes in the cell membrane (Fig. S5B). These results clearly indicate that NANOS2 can function independently of DDX6 in germ cells.

## Discussion

In this study, we introduced a method of chimera analysis to facilitate germ-cell-specific gene knockout studies. Although it is limited to genes functioning in a cell-autonomous manner, it can markedly shorten the experimental time. Currently, several gene editing techniques are available to quickly modify genes in ESCs, which makes chimera analyses more attractive. As we used the CRISPR/Cas9 technology for gene modification, any off-target modifications that were introduced in ES cells may have contributed to the observed phenotypes. However, we consider this unlikely because we also established a *Ddx6*-cKO mouse line using the same ES cells and confirmed all phenotypes observed in the chimera analyses. One problem of chimera analysis for germ cell studies is the possible disagreement between the sex of the host embryos and that of the ES cells. As we used XY-ES cells, embryos with a higher ES cell contribution became male. Therefore, this chimera method works well for the analysis of male germ cells in testes. However, we cannot use XY-ES cells for female germ cell analyses. Although we expected XX-ES cells to be replaced during the formation of ovaries, because somatic sex is determined by the Y chromosome and XY embryos cannot easily become female, we recommend XX host embryos for analyses of ovaries. Another matter of discussion is the choice of the driver for the inducible-Cre recombinase. We chose *Oct4-dPE* to drive CreER^T2^ because we have previously reported that this mouse line exhibited strong and specific Cre activity in germ cells upon TM injection^13^. Although the *Oct4* enhancer is active in ES cells, we did not expect Cre proteins to translocate to the nucleus in the absence of TM. However, we noted non-specific Cre activity during ES cell culture. We speculate that this is due to weak estrogen-like activity in the serum. This problem may be avoided by selecting a germ cell-specific enhancer lacking any activity in ES cells. We are currently searching for a more suitable enhancer.

We confirmed that *Ddx6*-KO led to P-body loss and the simultaneous loss of NANOS2 foci in germ cells even though NANOS2 was still abundant in the cytoplasm, indicating that the formation of NANOS2 foci is dependent on the DDX6. According to several previous reports on DDX6 function, DDX6 may not only be involved in P-body formation, but also in gene regulatory functions such as the storage of mRNA, nuclear exportation and promotion of translation^34,35^. Thus, it is possible that the different phenotypes were caused by varying DDX6 functions. To clarify whether disrupted P-body formation caused the loss of NANOS2 function, KO analyses of other P-body components will be useful. If these mutant germ cells, DDX6-null germ cells and NANOS2-null germ cells all have a similar phenotype, it will confirm that P-body formation is the major role of DDX6 for NANOS2 function.

P-body mediated RNA regulation is a critical step for regulating gene expression. As such, the P-body-mediated machinery regulates not only NANOS2 functions but also other developmental events, and differences between *Nanos2*-KO and *Ddx6*-cKO may be explained by global effects of P-body loss. We expect that the genes affected only in *Nanos2*-KO germ cells are involved in the phenotypes observed only in *Nanos2*-KO germ cells such as escaping from tubules. This phenotype may be due to the dedifferentiation of germ cells to migrating PGCs. Indeed, *Nanos2*-KO germ cells re-express a set of genes that are normally expressed in migrating PGCs, including *Nanos3, Sox2* and *Nanog*. However, these genes were up-regulated in both genotypes, ruling them out as the cause of germ cell escape. We found that many genes involved in membrane anchored proteins, membrane biogenesis and ion transports, are included for *Nanos2*-KO specific GO terms. We speculate that these genes are important to maintain cell membrane characteristics as male gonocytes.

This study also gave rise to an important question regarding the molecular mechanisms underlying NANOS2 functions. We previously reported that NANOS2 interacts with the CNOT-deadenylation complex, leading to the degradation of mRNA. Therefore, we speculated that most NANOS2 functions can be explained by P-body-dependent RNA destabilization. However, we observed DDX6-independent NANOS2 phenotypes, such as STRA8 up-regulation and escape from tubules, indicating that NANOS2 is still active without P-bodies. One explanation for this is the existence of a P-body-independent RNA destabilization mechanism because other P-body components involved in RNA degradation were still expressed in the *Ddx6*-cKO germ cells (Fig. 6b). In addition, NANOS2 can still interact with CNOT1 for RNA deadenylation and with DND1 for RNA interaction in the absence of DDX6^13^. It is also possible that NANOS2 has a role other than in RNA-decay because Drosophila Nanos was recently reported to stabilize target RNA in a 3′-UTR-dependent manner. A similar mechanism by which some targets are stabilized by NANOS2 may be conserved in mouse germ cells, and it may be independent of DDX6 and P-bodies. Further studies are required to address this possibility.

## Experimental Procedures

### Mice

Mice were housed in a specific pathogen-free animal care facility at the National Institute of Genetics (NIG). *mT/mG* mice (provided by Jackson Laboratory), *Oct4dPE-CreER*^*T2*^ (provided by A. Suzuki, Yokohama National University) and MCH (provided by CLEA Japan, Inc., Tokyo, Japan) mice were used in this study.

### Establishment of ES cell lines

Female *mT/mG* mice were superovulated by intraperitoneal injections of pregnant mare serum gonadotropin (PMSG) and human chorionic gonadotropin (hCG) at an interval of 48 h. hCG-injected female mice were mated with *Oct4dPE-CreER*^T2^ male mice, and the copulatory plug was confirmed the next morning. Blastocysts were collected from the uterus 3 days later. Collected blastocysts were plated on mitomycin-treated mouse embryonic fibroblast feeder cells in 2i-LIF medium (ESGRO Complete Basal medium; Millipore, Germany) containing leukemia inhibitory factor (LIF) (Wako, Tokyo, Japan), 0.4 µM MEK inhibitor PD0325901 (Wako, Tokyo, Japan), 3 µM GSK3 inhibitor CHIR99021 (Wako, Tokyo, Japan) and Penicillin-Streptomycin (Invitrogen). Outgrowths from blastocysts were disaggregated and passaged to new culture wells containing feeder cells in 2i-LIF medium. Once ES cell colonies emerged, these cells were expanded for genotyping and frozen. ES cells were treated with Proteinase K (Roche) and genotyped using the following primers: [WT Rosa locus; Rosa-F (5′-ctc tgc tgc ctc ctg gct tct-3′) and Rosa-R (5′-cga ggc gga tca caa gca ata-3′), *mT/mG* locus; Rosa-F and *mT/mG* R (5′-tca atg ggc ggg ggt cgt t-3′), and *CreER*^*T2*^; *CreER*^*T2*^-F (5′-gaa gca act cat cga ttg att tac gg-3′) and *CreER*^*T2*^-R(5′-tga agg gtc tgg tag gat cat act c-3′)]. We named the established ES cells TGOC referring to *Rosa-m****T****m****G****/****O****ct-dPE-****C****reERT*.

### Targeting strategies to produce *Ddx6*-cKO and *Nanos2*-KO ES cells

For construction of the *Ddx6* targeting vector, homology arms (LA; left arm and RA; right arm) and *Ddx6* exon5 were prepared by PCR from the mouse genome, and integrated into a vector containing two lox sequences via the sequential infusion method (The targeting vector sequence is available upon request). Cas9 target sites were selected using CRISPR direct (http://crispr.dbcls.jp/), and each target sequence was integrated into a modified px330 Cas9 vector (addgene) containing the pgk-puromycin cassette. These vectors were transfected into TGOC (XY) ESCs using Lipofectamine2000® (Invitrogen) and incubated with 1.5 µg/ml puromycin-containing medium for 2 days. Cloned ESCs were genotyped using the following primers: [*Ddx6-F* (5′-ttg tgc tgg gat gag cct a-3′) and *Ddx6-R* (5′-agt tgc atc aac gac agg aga g-3′)].

To generate *Nanos2*-KO ES cells, two Cas9 vectors were constructed and used for transfection. The *Nanos2*-null allele was detected by PCR using *Nanos2-F* (5′- ctg gga gat tgg agc cag cc-3′) and *Nanos2-R* (5′-ggt gtg agg tgt aga cgt gac g-3′) primers. The following are the cas9 guide sequences: *Ddx6* cas9 guide 1 (5′- cac tat gta cgc tgc atg aa-3′), *Ddx6* cas9 guide 2 (5′-ctg tgc agc caa act ccc cg-3′), *Nanos2* cas9 guide 1 (5′-gtc aaa ggg cgg tag gtc ca-3′) and *Nanos*2 cas9 guide 2 (5′-gtg ctt gca gaa gtt gca ta-3′).

### Production of chimeric mice

Eight-cell embryos collected by natural mating of MCH mice were treated with Tyrode’s solution to remove the zona pellucida. The embryos were aggregated with 4~10 ES cells and cultured in KSOM (Millipore, Germany) overnight. On the next day, embryos that developed into blastocysts or morulae were transplanted into the uterus of pseudopregnant foster mothers. To induce the CreER^T2^ activity, 500 µl of tamoxifen (Sigma-Aldrich, T5648) (10 mg/ml) was orally administered.

### Immunostaining

Embryonic gonads were fixed in 4% paraformaldehyde for 30 min at 4 °C. Gonads were next submerged in 10 and 20% sucrose in PBS for 1 h each at 4 °C, and in 30% sucrose in PBS overnight at 4 °C. The gonads were then embedded in Tissue-Tek O.C.T. compound (Sakura Finetek, Tokyo, Japan), and frozen in liquid nitrogen. Six-micrometer thick sections of each gonad were applied to glass slides, and autoclaved in citrate buffer. After pre-incubation with 3% skim milk in PBS-T (PBS with 0.1% Tween20 (Sigma-Aldrich)) at room-temperature for 30 min, the sections were reacted with primary antibodies overnight at 4 °C at the following dilutions: anti-MVH (1:1000, abcam), anti-GFP (1:1000, aves), anti-Rck/p54 (1:300, MBL), anti-DCP1A (1:300, abnova), rabbit anti-NANOS2 (1:100), mouse anti-NANOS2 (1:200), TRA98 (1:4000, a gift from Y. Nishimune, Osaka University), anti-DAZL (1:200, a gift from A. Suzuki, Yokohama National University), anti-DNMT3L (1:500, a gift from S. Yamanaka, iCeMS, Kyoto University), anti-pH3 (1:200, millipore), anti-NANOS3 (1:200), anti STRA8 (1:200, Abcam) and anti-laminin (1:3000, Sigma-Aldrich). Secondary antibodies labelled with Alexa Fluor 488, 594 or 647 (1:1000, Invitrogen) and Alexa 488 (1:1000, Jackson ImmunoResearch) were used. DNA was counter-stained with DAPI (100 ng/ml). Fluorescence microscopy was performed using Olympus FV1200, and images were processed with ImageJ.

### Germ cell collection

To collect mutant germ cells, single-cell suspensions were prepared from 3–9 gonads from 15 dpa chimera embryos by incubation with 0.15% trypsin-EDTA at 37 °C for 5–10 min, and filtered through a 35-µm sieve (BD BioScience, San Jose, CA). The mutant germ cells were sorted by a JSAN Desktop Cell Sorter using the GFP fluorescein signal.

### RNA-Seq analysis

For RNA-Seq analysis, we prepared RNA from 2,500–20,000 sorted germ cells using the RNeasy Micro Kit (Qiagen). Twenty percent of the purified RNA was used for amplification with the TargetAmp 1-Round aRNA Amplification Kit 103 (epicenter, WI, USA). The amplified RNA was used for cDNA library construction using the KAPA Stranded mRNA-Seq Kit (KAPA, NIPPON genetics, JAPAN). Two independent cDNA libraries were prepared for each group and sequenced on the Illumina HiSeq 3000 platform to obtain 36-base single reads. Reads were aligned to the mouse genome (mm10) using Bowtie^34^, and gene expression changes were analyzed by DESeq 2^35^. During the sequence read quality check, the quality of one *Ddx6*-cKO sample was found to be too low. Thus, we excluded this sample from further analyses.

### GO and pathway analysis

To characterize the major differences among the samples, we utilized the Gene Ontology (GO) and pathway analyses via the DAVID tool (https://david.ncifcrf.gov/)^36^. For visualization of GO results, we used the R package GOplot^33^.

### Statistical analysis

Statistical significance between genotypes was assessed by the Student’s *t*-test. Error bars represent the s.d.

### Ethical approval

The research reported involves mice. All mice were handled and propagated in accordance with National Institute of Genetics (NIG) guidelines, and all experimental procedures were approved by the Committee for Animal Care and Use in National Institute of Genetics.

## Electronic supplementary material

Supplementary Information

Table S1

Table S2

Table S3

## Data Availability

All data generated during this study except for RNA-seq are included in this article (and its Supplementary Information Files). Sequence data have been submitted to DNA Data Bank of Japan under accession number DRA007505.
