# Supplementary material for: ES-mediated chimera analysis revealed requirement of DDX6 for NANOS2 localization and function in mouse germ cells
Source: Sci Rep. 2019 Jan 24;9:515. doi: 10.1038/s41598-018-36502-0 (PMC6345806; doi:10.1038/s41598-018-36502-0)
Supplement: Supplementary file 1 — Supplementary Information [file 41598_2018_36502_MOESM1_ESM.pdf]

Supplementary Information

ES-mediated chimera analysis revealed requirement of DDX6 for NANOS2 localization and function in mouse germ cells

Ryuki Shimada, Makoto Kiso and Yumiko Saga

Contents:

Supplementary Figure S1-S5
Source data of Figure 2C

Fig.S1

ES #	Aggregated	Transplanted blastocysts	Dissected at	Embryos recovered	ES contribution		
					0 %	10-50%	50-100%
TGOC#8	22	22	E14.5	5	0	1	4
TGOC#8	56	50	E16,5	15	2	1	12

#	Stage	Chimerism	Gonad
M1	E14.5	100	Testis
M2	E14.5	30	Testis
M3	E14.5	100	Testis
M4	E14.5	100	Testis
M5	E14.5	100	Testis

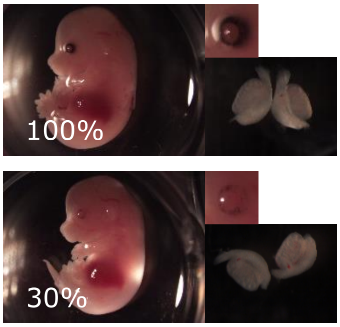

#	Stage	Chimerism	Gonad
M1	E16.5	70	Testis
M2	E16.5	100	Testis
M3	E16.5	70	Testis
M4	E16.5	60	Testis
M5	E16.5	80	Testis
F1	E16.5	10	Ovary
M6	E16.5	80	Testis
M7	E16.5	0	Testis
M8	E16.5	100	Testis
M9	E16.5	100	Testis
M10	E16.5	100	Testis
M11	E16.5	0	Testis
M12	E16.5	80	Testis
M13	E16.5	90	Testis
M14	E16.5	90	Testis

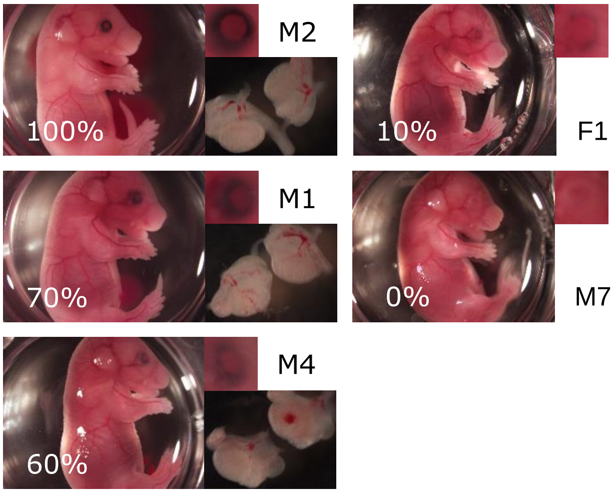

Figure S1. The efficiency of chimera produced with TGOC ES cells. Results for chimera production with parental TGOC ESCs. Stage, chimerism and gonadal type are shown. Chimerism was inferred from eye color. Gonadal types were morphologically determined. Representative embryo images are also shown. An E16.5 M2 chimera is shown in Fig.1E and F. M: male and F: female

Fig.S2

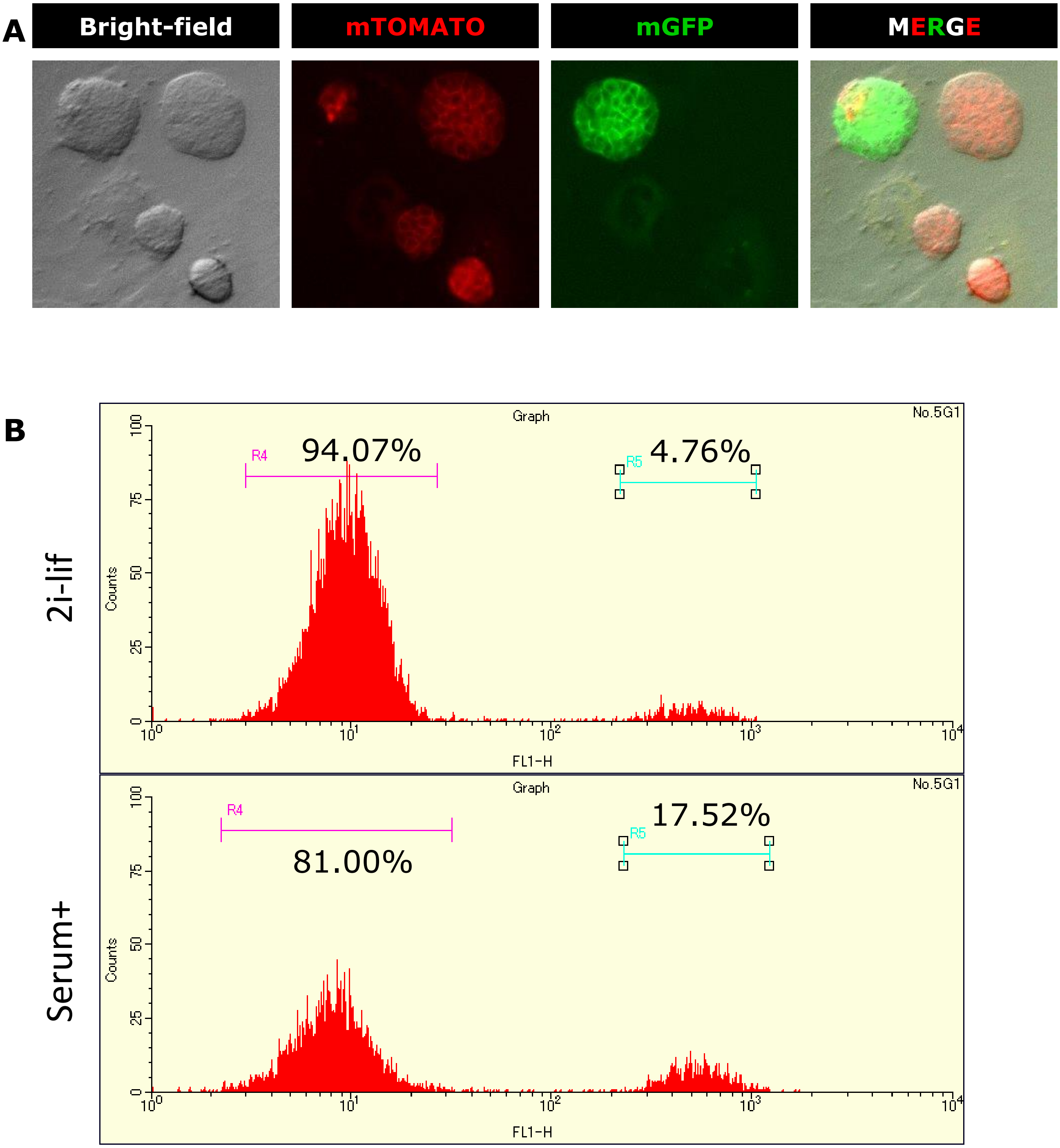

Figure S2. TGOC ESCs express mTOMATO or mGFP
(A) Expression of membrane anchored fluorescent proteins in TGOC ECSs cultured in 2i-Lif without TM. There are some clones expressing mGFP among the mTOMATO-positive cells. (B) Population differences between 2i-LIF and serum+ culture conditions. The horizontal axis indicates intensity of GFP and the vertical axis indicates cell number.

Fig.S3

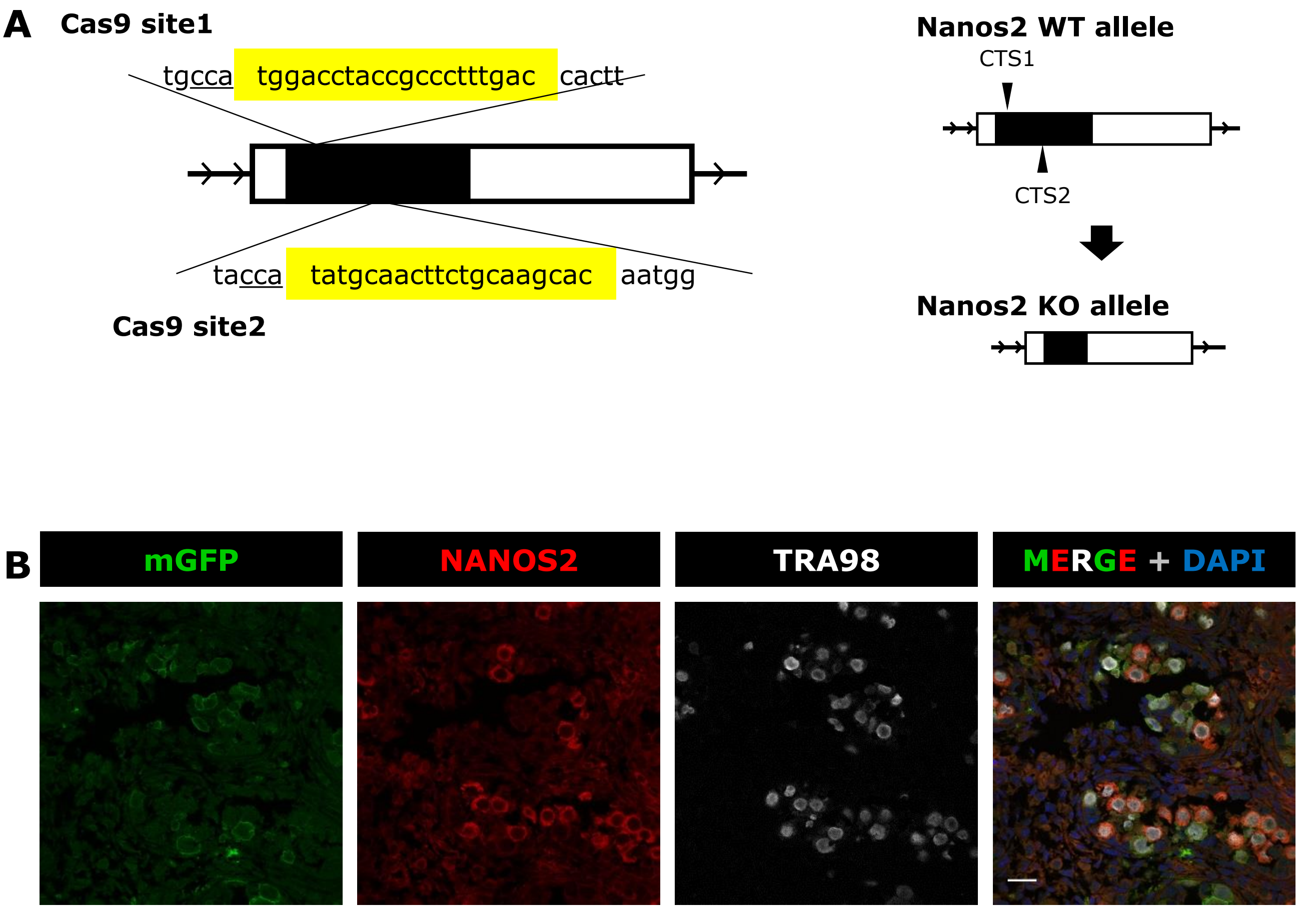

Figure S3. KO strategy for *Nanos2* using the Cas9-mediated method. (A) Two Cas9 target sites were designed to eliminate part of the *Nanos2* CDS. CTS: Cas9 Target Site. White boxes indicate the non-CDS and black boxes indicate the CDS. The mutation was introduced in the same TGOC line used for generating *Ddx6*-cKO lines. (B) Immunofluorescence image of chimeric testes produced by *Nanos2*-KO ESCs. mGFP-positive (green)TRA98-positive (white) germ cells lost NANOS2 (red) signal (arrowheads). TM was administered at 11 dpa (E12.5) and embryos were prepared at 15 dpa (E16.5). Scale bars indicate 20 μm.

FigS4

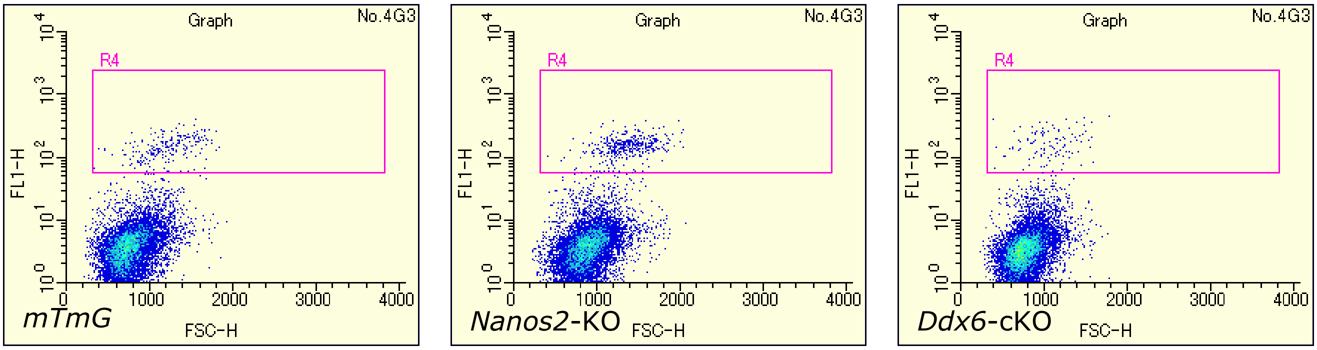

Figure S4. FACS plot for germ cell collection
For RNA-seq, germ cells were prepared from embryonic chimeric testes produced with TGOC without TM, TGOC/*Nanos2*-KO with TM and TGOC/*Ddx6*-cKO with TM. Each plot shows the GFP intensity on the vertical axis. Purple boxes indicate the sorted GFP-positive population.

FigS5

A. Common DEGs

ID	Description	Up	Dn	genes
GO:0043646	anatomical structure formation involved in morphogenesis	31	49	*Tet1*, *C3*, *Nme8*, *Ccdc113*
GO:0055001	muscle cell development	6	16	
GO:0048468	cell development	44	74	*Lefty1*, *Sox2*, *Mt2*
GO:0055002	striated muscle cell development	6	14	
GO:0070206	protein trimerization	3	8	
GO:0048598	embryonic morphogenesis	25	20	*Sox2*
GO:0030030	cell projection organization	16	62	*Nme8*, *Ccdc113*
GO:0032989	cellular component morphogenesis	25	52	*Ccdc113*
GO:0001704	formation of primary germ layer	10	6	*Sox2*
GO:0048747	muscle fiber development	2	9	

B. NANOS2-specific DEGs

ID	Description	Up	Dn	genes
GO:0006811	ion transport	66	174	*Hpn*, *Slc51a*, *Vdr*, *Enpp1*
GO:0006796	phospho-containing compound metabolic process	175	257	*Plaur*, *Hpn*, *Upp1*, *Enpp1*, *Cap2*, *Adgrg6*, *Dyrk4*
GO:0006793	phosphorus metabolic process	175	257	*Plaur*, *Hpn*, *Upp1*, *Enpp1*, *Cap2*, *Adgrg6*, *Dyrk4*
GO:007155	cell adhesion	77	181	*Chl1*
GO:0051049	regulation of transport	97	194	*Vdr*, *Enpp1*, *Ahsg*
GO:0022610	biological adhesion	77	182	*Chl1*
GO:0022607	cellular component assembly	174	196	*Ahsg*
GO:0048468	cell development	126	225	*Stra8*, *Chl1*, *Hpn*
GO:0030001	metal ion transport	43	100	*Hpn*, *Vdr*, *Adgrg6*
GO:0006928	movement of cell or subcellular component	73	202	*Chl1*

C. DDX6-specific DEGs

ID	Description	Up	Dn	genes
GO:0010468	regulation of gene expression	67	41	*Otx2*, *Etv5*, *Lhx1*, *Prdm14*
GO:0045165	cell fate commitment	13	4	*Otx2*, *Prdm14*
GO:0019219	regulation of nucleobase-containing compound metabolic process	63	38	Otx2, Etv5, *Lhx1*, *Prdm14*
GO:0051252	regulation of RNA metabolic process	60	34	*Otx2*, *Etv5*, *Lhx1*, *Prdm14*
GO:0035456	response to interferon-beta	8	0	
GO:2001141	regulation of RNA biosynthetic process	58	33	*Otx2*, *Etv5*, *Lhx1*, *Prdm14*
GO:0006351	transcription, DNA-templated	53	31	*Otx2*, *Etv5*, *Lhx1*, Prdm14
GO:0006355	regulation of transcription, DNA-templated	57	33	*Otx2*, *Etv5*, *Lhx1*, *Prdm14*
GO:1903506	regulation of nucleic acid-templated transcription	57	33	*Otx2*, *Etv5*, *Lhx1*, *Prdm14*
GO:0006366	transcription from RNA-polymerase II promoter	34	19	*Otx2*, *Etv5*, *Lhx1*, *Prdm14*

Figure S5. GO analysis for DEGs

The top 10 GO terms (biological processes) for common (A), *Nanos2*-KO-specific (B) and *Ddx6*-KO-specific (C) DEGs were presented. The gene names shown in Fig. 6D-F are indicated as assigned.

Source image

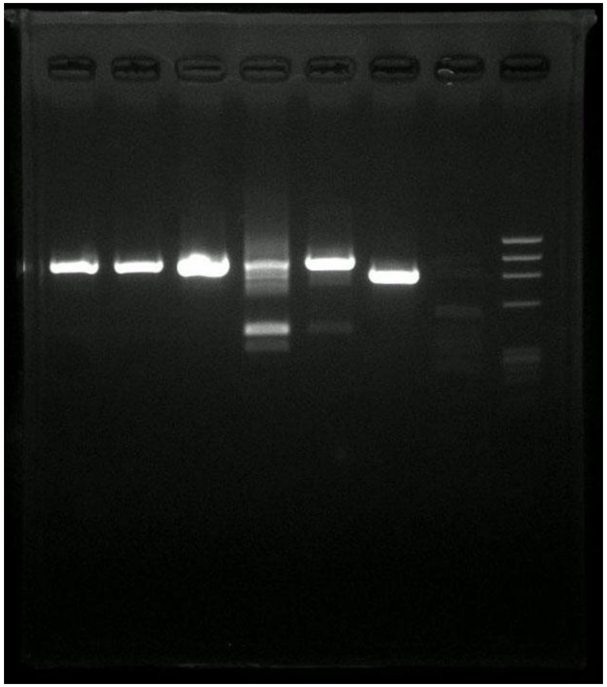

Source image of Fig.2C
